# Assessment of G Protein-Coupled Oestrogen Receptor Expression in Normal and Neoplastic Human Tissues Using a Novel Rabbit Monoclonal Antibody

**DOI:** 10.3390/ijms23095191

**Published:** 2022-05-06

**Authors:** Maria Bubb, Anna-Sophia Lieselott Beyer, Pooja Dasgupta, Daniel Kaemmerer, Jörg Sänger, Katja Evert, Ralph M. Wirtz, Stefan Schulz, Amelie Lupp

**Affiliations:** 1 Institute of Pharmacology and Toxicology, Jena University Hospital, 07747 Jena, Germany; maria.bubb@gmx.de (M.B.); anna-sophia.beyer@uni-jena.de (A.-S.L.B.); pooja.dasgupta@med.uni-jena.de (P.D.); stefan.schulz@med.uni-jena.de (S.S.); 2 Department of General and Visceral Surgery, Zentralklinik Bad Berka, 99438 Bad Berka, Germany; daniel.kaemmerer@zentralklinik.de; 3 Laboratory of Pathology and Cytology Bad Berka, 99438 Bad Berka, Germany; pathologie.bad-berka@t-online.de; 4 Department of Pathology, University of Regensburg, 93053 Regensburg, Germany; katja.evert@klinik.uni-regensburg.de; 5 Institute of Pathology, University Medicine of Greifswald, 17475 Greifswald, Germany; 6 STRATIFYER Molecular Pathology GmbH, 50935 Cologne, Germany; ralph.wirtz@stratifyer.de

**Keywords:** G-protein-coupled oestrogen receptor, GPER, GPR30, antibody, immunohistochemistry, neuroendocrine, tumours

## Abstract

In addition to the classical oestrogen receptors, ERα and ERβ, a G protein-coupled oestrogen receptor (GPER) has been identified that primarily mediates the rapid, non-genomic signalling of oestrogens. Data on GPER expression at the protein level are contradictory; therefore, the present study was conducted to re-evaluate GPER expression by immunohistochemistry to obtain broad GPER expression profiles in human non-neoplastic and neoplastic tissues, especially those not investigated in this respect so far. We developed and thoroughly characterised a novel rabbit monoclonal anti-human GPER antibody, 20H15L21, using Western blot analyses and immunocytochemistry. The antibody was then applied to a large series of formalin-fixed, paraffin-embedded human tissue samples. In normal tissue, GPER was identified in distinct cell populations of the cortex and the anterior pituitary; islets and pancreatic ducts; fundic glands of the stomach; the epithelium of the duodenum and gallbladder; hepatocytes; proximal tubules of the kidney; the adrenal medulla; and syncytiotrophoblasts and decidua cells of the placenta. GPER was also expressed in hepatocellular, pancreatic, renal, and endometrial cancers, pancreatic neuroendocrine tumours, and pheochromocytomas. The novel antibody 20H15L21 will serve as a valuable tool for basic research and the identification of GPER-expressing tumours during histopathological examinations.

## 1. Introduction

The classical oestrogen receptors, ERα and ERβ, were identified in 1962 and 1996, respectively, and predominantly mediate genomic signalling. In the 2000s, a third receptor, G protein-coupled oestrogen receptor (GPER), also known as GPR30, was identified and recognised as a critical mediator of rapid signalling in response to oestrogens. GPER belongs to the large family of 7-transmembrane domain G protein-coupled receptors and has been reported to couple with both Gs and Gi/o proteins [1]. Hence, downstream pathways induced by GPER activation include (Gs protein-mediated) stimulation of adenylate cyclase, leading to increased cAMP concentrations, protein kinase A activation, and decreased activation of extracellular signal-regulated kinases 1/2 (ERK1/2) [2], but also (via Gi/o proteins) Src-mediated stimulation of metalloproteinase activity, which is associated with the release of the heparin-binding epidermal growth factor (EGF) and the subsequent transactivation of EGF receptor (EGFR). This in turn leads to activation of ERK1/2 and the phosphoinositide 3-kinase (PI3K)/Akt axis [3]. Subsequently produced phosphatidyl-3,4,5-trisphoshate can then lead to activation of the transcription factor SF-1, eventually resulting in elevated aromatase expression, increased oestradiol synthesis, and cell proliferation [4]. GPER also mediates an increase in the activity of endothelial nitric oxide (NO) synthase (eNOS), which results in NO production and consecutive vasodilation [5,6]. GPER can further lead to an increase in the activity of sphingosine kinase, resulting in the production of sphingosine-1-phosphate by cancer cells [7]. In addition, GPER regulates calcium mobilisation and potassium channel activity [8,9,10,11]. Finally, GPER modulates gene expression of, e.g., c-fos and the cyclins A, D1 or E through a mechanism distinct from those mediated by ERα or ERβ [1,12,13,14,15]. Through all these signalling pathways, GPER is involved in the regulation of a wide range of cellular functions, such as cell growth, proliferation, migration, and apoptosis, and exerts an influence on the endocrine, nervous, immune, and cardiovascular system as well as cancer development [1].

A large number of synthetic and natural oestrogenic compounds have been shown to interact with GPER. GPER agonists include natural oestrogens, such as 17β-oestradiol and 2-methoxy-oestradiol; selective oestrogen receptor modulators (SERMs), such as tamoxifen, 4-hydroxytamoxifen, and raloxifene; selective oestrogen receptor down-regulators (SERDs), such as fulvestrant; pesticides; plastic compounds that act as endocrine disruptors, including atrazine, bisphenol A, zearalenone, nonylphenol, and kepone; and numerous phytoestrogens, including genistein, quercetin, equol, and resveratrol. GPER antagonists include synthetic compounds, such as MIBE, and the natural oestrogens 2-hydroxy-oestradiol and oestriol [1,15]. In addition to these substances, which have overlapping affinities for all three oestrogen receptors, highly selective ligands have been synthesised for GPER, including the agonist G-1 and the antagonists G-15 and G-36 [15,16,17,18].

Studies on GPER expression in normal human tissues are scarce, especially at the protein level, likely because highly specific monoclonal antibodies are not yet available for this receptor. However, this receptor has been characterised in GPER-LacZ reporter mice, through reverse transcriptase quantitative polymerase chain reaction (RT-qPCR), and Western blot analyses using polyclonal anti-GPER antibodies, revealing GPER expression in the cortex, anterior pituitary, pancreatic islets, adrenal medulla, gastric chief cells, and kidney in mice. The receptor has been demonstrated to be involved in the regulation of pancreatic insulin, glucagon, and somatostatin secretion, and to modulate insulin responsiveness in the peripheral tissues of mice [19,20,21].

GPER-knockout mice show visceral obesity, increased low-density lipoprotein cholesterol levels, insulin resistance, hyperglycaemia, reduced glucose tolerance, generalised inflammation, increased blood pressure, cardiovascular dysfunction, decreased skeletal development leading to reduced body growth, diminished serum insulin-like growth factor 1 levels, and impaired T cell production; however, in contrast to ERα-knockout mice, GPER-knockout mice display no reduction in fertility [19,20,22,23,24]. Correspondingly, treatment of ovariectomised female mice using the selective GPER agonist G-1 reduced body weight, diminished body fat content, improved glucose homeostasis, increased energy expenditure, reduced fasting cholesterol, glucose, and insulin levels, and decreased the expression of inflammatory markers [25]. Thus, targeting GPER may represent an interesting therapeutic approach to common diseases, including obesity, diabetes, hypertension, atherosclerosis, myocardial infarction, and stroke [1,15].

Studies of GPER in human tumours have primarily focused on sex hormone-dependent cancers, with contradictory results, which may in part be due to the wide variety of polyclonal antibodies from commercial and non-commercial sources that have been used in these studies.

We have developed a novel rabbit monoclonal antibody, 20H15L21, targeted against the carboxyl-terminal tail of human GPER. In the present study, we demonstrate that this antibody is well-suited for both basic research applications, such as Western blot and immunohistochemical staining during routine histopathological examinations. To obtain a broad profile of GPER expression throughout the human body and in a wide range of human tumours, the antibody was applied to a large series of formalin-fixed, paraffin-embedded normal and neoplastic human tissue samples, revealing, amongst others, GPER expression in pancreatic islet cells and pancreatic neuroendocrine tumours. Based on these findings, we expanded the number of neuroendocrine tumours examined and evaluated GPER expression in a broad panel of bronchopulmonary (BP-NENs) and gastroenteropancreatic neuroendocrine neoplasms (GEP-NENs) of different origins. The staining results were then correlated with clinical data, such as tumour grading, tumour staging, and overall patient survival.

## 2. Results

### 2.1. Characterisation of the Rabbit Monoclonal Anti-Human GPER Antibody 20H15L21

**Figure 1:**
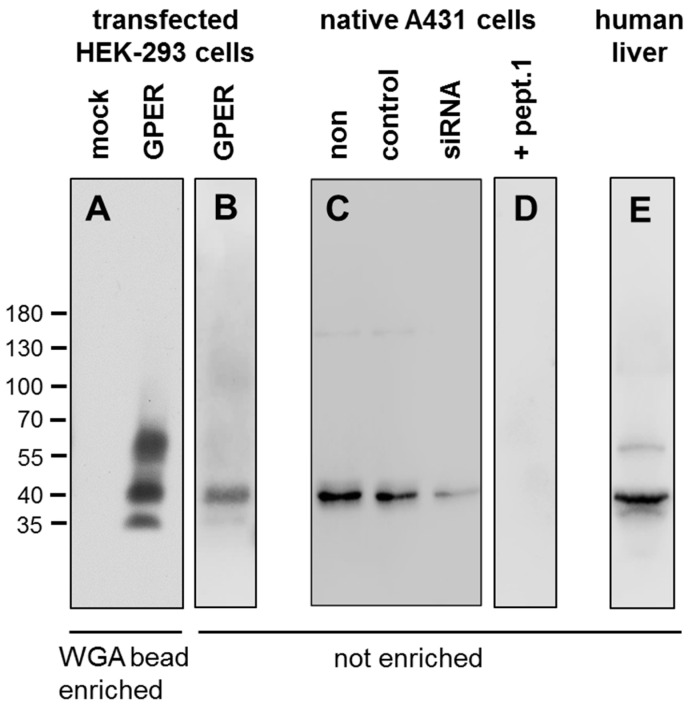
Characterisation of the novel rabbit anti-human GPER antibody 20H15L21 by Western blot analysis. Western blot analysis of whole-cell preparations from mock- or stably GPER-transfected HEK-293 cells (**A**,**B**), A321 cells (**C**,**D**) or normal human liver (**E**). GPER receptors were either enriched using wheat germ lectin agarose (WGA) beads (**A**) or not enriched (**B**–**E**). Samples were separated by 7.5% sodium dodecyl sulfate-polyacrylamide gel electrophoresis and blotted onto polyvinylidene difluoride membranes. The membranes were then incubated with 20H15L21, and the blots were developed using enhanced chemiluminescence. The relative migration of protein molecular weight markers is indicated (in kDa). Note that the novel anti-GPER antibody 20H15L21 selectively detected only the targeted GPER receptor and did not cross-react with other membrane proteins. (**C**) For further analysis of the specificity of the novel antibody, GPER expression was silenced in A431 cells endogenously expressing GPER using a GPER-specific siRNA (siRNA). Here, a distinct reduction of the immunosignal after incubation of the cells with the specific siRNA in comparison with non-transfected cells (non) or those transfected with the empty vector (control) was noted. (**D**) For an additional adsorption control, the anti-GPER antibody was preincubated for 2 h with 10 µg/mL of the peptide used for the immunisation of the rabbit (+ pept.1). Here, a complete extinction of the immunosignal was observed. Representative results from one of three independent experiments are shown.

The specificity of the anti-human GPER antibody 20H15L21 was first tested by Western blot analysis. When wheat germ lectin agarose (WGA) bead-enriched preparations from stably GPER-transfected HEK-293 cells were electrophoretically separated and immunoblotted, the antibody recognised broad bands migrating at approximately 35, 40, and 55–70 kDa (Figure 1A, right panel), which corresponds well with the expected molecular weights of the different glycosylated species as well as a degraded form of the receptor in GPER overexpressing cells [26]. By contrast, no immunosignal was observed in mock-transfected HEK-293 cells, which do not endogenously express GPER (Figure 1A, left panel). When non-enriched preparations of GPER-transfected HEK-293 cells or A431 cells endogenously expressing GPER were electrophoretically separated, only a single band at approximately 40 kDa could be detected (Figure 1B,C, left panel), whereas in preparations of normal human liver again three bands were noted (Figure 1E). When GPER expression was silenced in A431 cells using a GPER-specific siRNA, the immunosignal was distinctly reduced in comparison with non-transfected cells or those transfected with an empty vector (Figure 1C, right panel). Furthermore, after preincubating 20H15L21 for 2 h with 10 µg/mL of the peptide used for the immunisation of the rabbit (peptide 1), a complete extinction of the immunosignal was observed (Figure 1D), whereas no effect was noted after preincubating the antibody with peptide 2, which consisted of an amino acid sequence found in another region of the receptor (data not shown).

**Figure 2:**
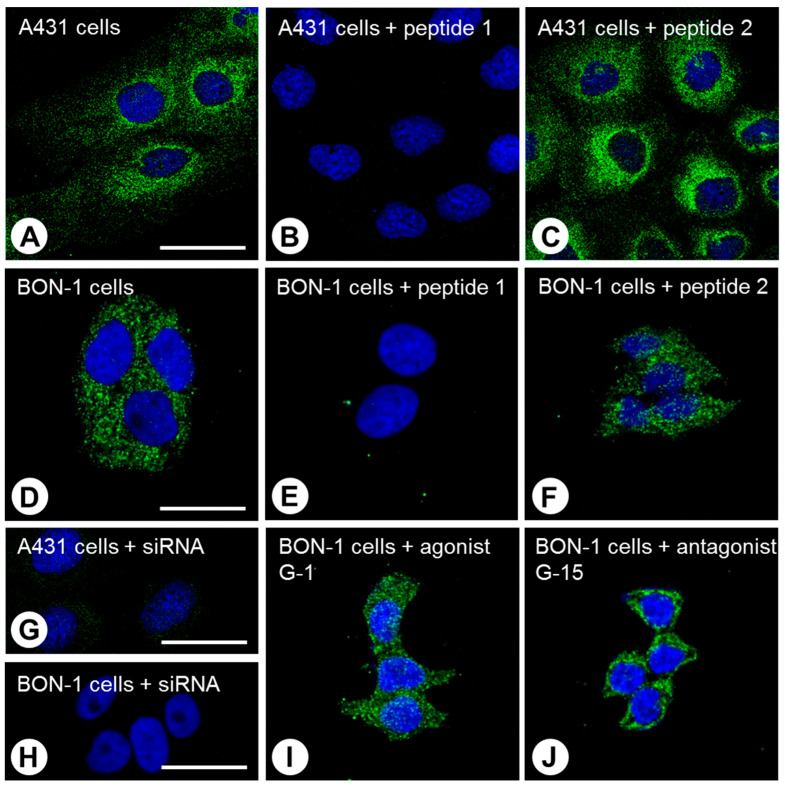
Immunocytochemical analysis of A431 cells and BON-1 cells endogenously expressing GPER. A431 cells (**A**–**C**,**G**) and BON-1 cells (**D**–**F**,**H**–**J**) were fixed and stained with the novel anti-GPER antibody 20H15L21, followed by an Alexa Fluor 488-conjugated anti-rabbit secondary antibody. For adsorption controls, the anti-GPER antibody was preincubated for 2 h with either 10 µg/mL of the peptide used for the immunisation of the rabbit (peptide 1; **B**,**E**) or with a peptide representing an amino acid sequence at the N-terminal region of the receptor (peptide 2; **C**,**F**). Note that after preincubation with peptide 1 but not peptide 2, a complete extinction of the immunosignal was observed. For further analysis of the specificity of the novel antibody, GPER expression was silenced in A431 and BON-1 cells using a GPER-specific siRNA. Again, a complete disappearance of the immunosignal was noted (**G**,**H**). In an additional set of experiments, BON-1 cells were exposed to either 10 µM of the selective GPER agonist G-1 or 10 µM of the selective GPER antagonist G-15 for 30 min. After agonist stimulation, intracellular distribution was retained (**I**), whereas treatment with the antagonist resulted in the redistribution of GPER to the plasma membrane (**J**). Green, GPER immunosignal; blue, DAPI nuclear staining. Representative results from one of three independent experiments are shown. Scale bars: (**A**–**C**,**G**) 40 µm; (**D**–**F**,**H**–**J**) 20 µm. GPER, G protein-coupled oestrogen receptor; DAPI, 4′,6-diamidino-2-phenylindole.

The anti-GPER antibody was further characterised using immunofluorescent staining of GPER-HEK-293 cells, or BON-1 and A431 cells, which express endogenous GPER. In all cases, mainly a punctate, cytoplasmic expression pattern was observed for GPER (Figure 2A,D). After preincubating 20H15L21 with the immunising peptide (peptide 1), the immunosignal was completely abolished (Figure 2B,E), whereas no change in immunosignal was observed after preincubating the antibody with peptide 2 (Figure 2C,F). When GPER expression in A431 or BON-1 cells was silenced using a GPER-specific siRNA, no immunosignal was observed (Figure 2G,H). After incubating the cells with the specific GPER agonist G-1, no major changes were observed in the receptor distribution (Figure 2I); by contrast, after treatment with the specific GPER antagonist G-15, the immunosignal became predominantly localised at the plasma membrane (Figure 2J).

### 2.2. Immunohistochemical Detection of GPER Expression in Normal Human Tissues

**Figure 3:**
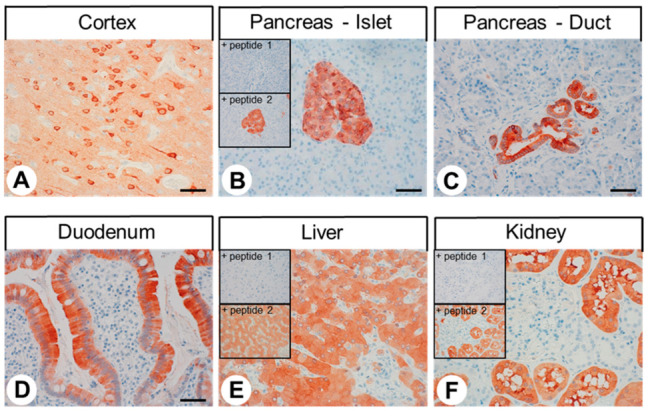
Immunohistochemical detection of GPER localisation in different normal human tissues. Immunohistochemical staining (red-brown colour) and counterstaining with haematoxylin. Scale bar: 100 µm (**A**–**F**). Insets in (**B**,**E**,**F**) represent adsorption controls, in which the anti-GPER antibody 20H15L21 was preincubated for 2 h with either the peptide used to immunise the rabbit (+ peptide 1) or a peptide representing an amino acid sequence at the N-terminal region of the receptor (+ peptide 2).

The rabbit monoclonal anti-GPER antibody 20H15L21 was used to perform immunohistochemical staining of various normal human tissues. In all cases, both cytoplasmic and membranous staining patterns were observed. A set of both normal and neoplastic tissues showing positive GPER staining was also subjected to immunoadsorption experiments using either the immunising peptide (peptide 1) or a peptide corresponding to another region of the receptor (peptide 2). In all cases, preincubation of the antibody with peptide 1 led to the complete extinction of the immunosignal, whereas no changes in the immunostaining outcomes were observed after the preincubation of 20H15L21 with peptide 2 (see insets in Figure 3B,E,F and Figure 5E). Serial sections of GPER-positive and -negative normal and neoplastic tissue samples were also stained with the polyclonal rabbit anti-GPER antibody ab39742 (Abcam, Cambridge, UK), which is directed against the C-terminal tail of GPER as well, or with the polyclonal goat anti-GPER antibody AF5534 (R&D systems, Minneapolis, MN, USA), which is directed against the N-terminal tail of GPER, and thus, another epitope of the receptor. These additional experiments revealed similar staining results when compared to those obtained with the novel monoclonal antibody 20H15L21. However, both polyclonal antibodies, especially ab39742, detected less GPER receptors in the GPER-positive tissues and caused a higher amount of non-specific nuclear and background staining in comparison with 20H15L16 (see Supplementary Figures S1–S3). Serial sections from the immunohistochemistry slides of ten selected tumour samples were also subjected to qRT-PCR analysis of GPER mRNA isoforms 2 and 3 + 4, which have an identical coding region and differ only in the 5′ untranslated region. These investigations revealed an interrelationship between GPER mRNA values and the IRS values obtained with the novel antibody 20H15L21. However, most probably due to the small number of samples evaluated, these correlations did not reach significance (IRS vs. dCT mRNA isoform 2, rsp = 0.741, *p* = 0.092; IRS vs. dCT mRNA isoforms 3 + 4, rsp = 0.617, *p* = 0.192). It should also be noted that the mRNA analyses were performed on entire tumour sections, while calculation of IRS values was only applied to the staining of tumour cells and not the (mostly negative) tumour stroma (Supplementary Figures S2 and S3).

**Figure 4:**
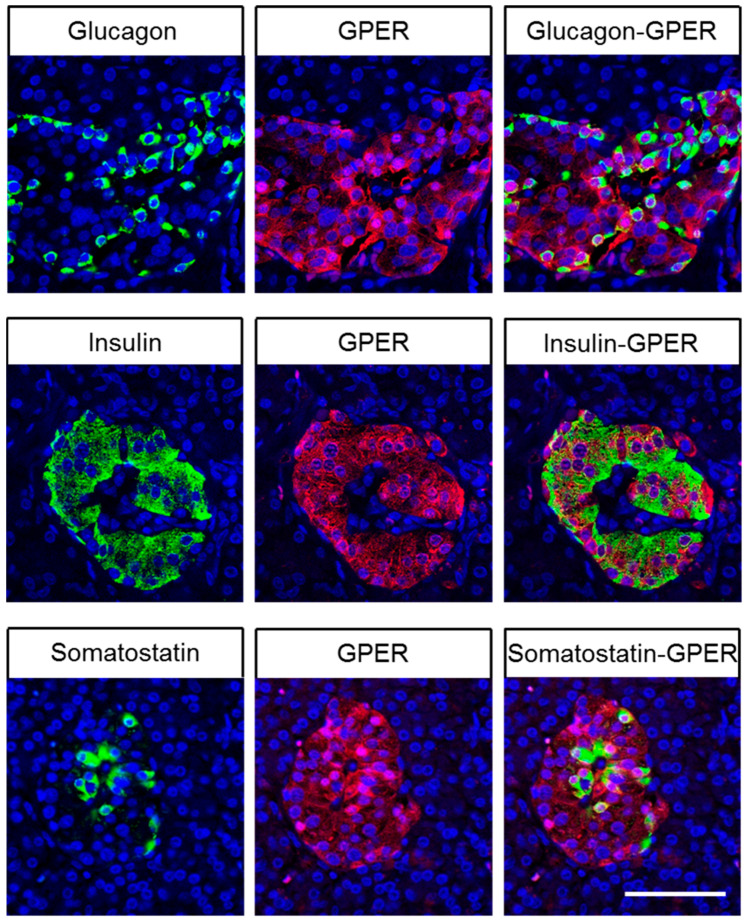
Double-labelling immunohistochemical analysis of human pancreatic islets. Sections were dewaxed and microwaved in citric acid. Adjacent sections of each tissue sample were incubated with rabbit monoclonal anti-GPER antibody 20H15L21 together with mouse monoclonal antibodies against glucagon or insulin, or a rat antibody against somatostatin-14/28. Labelling for GPER was visualised using a Cy3-conjugated anti-rabbit antibody (red), and labelling for insulin, glucagon, or somatostatin-14/28 was visualised using an Alexa Fluor 488-conjugated anti-mouse or anti-rat antibody (green). Scale bar: 100 µm.

As depicted in the examples shown in Figure 3, very strong GPER immunostaining was detected in the pyramidal cells of layers II and V of the cortex. Throughout all layers of the cortex, the neuropil and synapses were also slightly GPER-positive. GPER was also present in a subpopulation of cells within the anterior pituitary, in pancreatic islets, pancreatic ducts, the base of the fundic glands in the stomach, the epithelium and Brunner’s glands of the duodenum, the epithelium of the gallbladder, hepatocytes in the liver, the proximal tubules and Bowman’s capsule of the glomeruli in the kidney, the adrenal medulla, and syncytiotrophoblasts and decidua cells of the placenta. Occasionally, capillaries and the endothelia of small arteries were also GPER-positive. By contrast, no immunostaining was observed in the posterior pituitary, thyroid gland, lung, heart, tonsils, thymus, spleen, lymph nodes, neuroendocrine cells and intramural ganglia of the gut, acinar cells of the exocrine pancreas, normal prostate, testicular, breast, ovarian, or uterine tissue, bone, or bone marrow. To more precisely differentiate GPER-positive cells in pancreatic islets, additional double-labelling experiments were performed to identify cells expressing glucagon, insulin, or somatostatin-14/28, which clearly revealed the presence of GPER in glucagon-secreting alpha cells, insulin-producing beta cells, and somatostatin-positive delta cells (Figure 4).

### 2.3. Immunohistochemical Detection of GPER Expression in Different Human Tumour Entities

**Figure 5:**
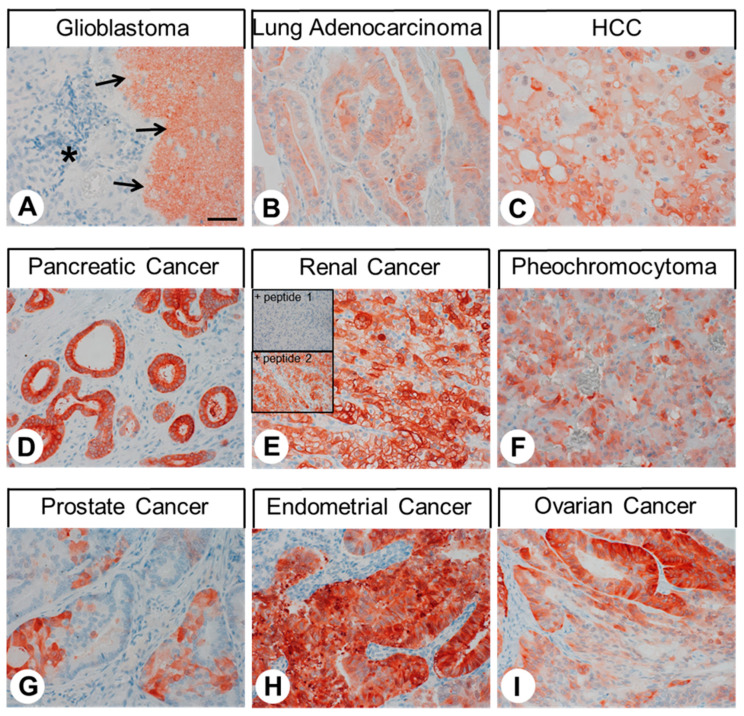
Immunohistochemical detection of GPER localisation in different human tumour entities. Immunohistochemical staining (red-brown colour) and counterstaining with haematoxylin. Scale bar: 100 µm (**A**–**I**). Insets in E represent adsorption controls, in which the anti-GPER antibody 20H15L21 was preincubated for 2 h with either the peptide used to immunise the rabbit (+ peptide 1) or with a peptide representing an amino acid sequence at the N-terminal region of the receptor (+ peptide 2). Asterisk in (**A**), tumour; arrows in (**A**), surrounding GPER-positive non-neoplastic cortical tissue.

The GPER distribution patterns observed in human tumour samples are summarised in Table 1. Additionally, representative examples of immunostaining are shown in Figure 5. Similar to the outcomes for normal tissues, both cytoplasmic and membranous staining was observed. As evidenced by the minimum and maximum IRS values assigned to the individual tumours within the various tumour types, and as indicated in Figure 5G,I, GPER expression displayed substantial inter- and intra-individual variability. Often, strong staining was observed only in small areas of the tumours, whereas some large regions of the samples were completely GPER-negative, resulting in a low overall rating. Pronounced GPER expression, including a higher number of GPER-positive cases (IRS ≥ 3) and higher IRS values, was particularly prevalent in hepatocellular carcinomas, pancreatic adenocarcinomas, renal clear cell carcinomas, pheochromocytomas, and endometrial cancer. However, for some other tumour entities, such as lung adenocarcinomas, neuroendocrine tumours, and ovarian cancer, individual cases with high GPER expression rates were also observed.

### 2.4. GPER Expression in Bronchopulmonary and Gastroenteropancreatic Neuroendocrine Tumours

#### 2.4.1. GPER Expression Pattern

**Figure 6:**
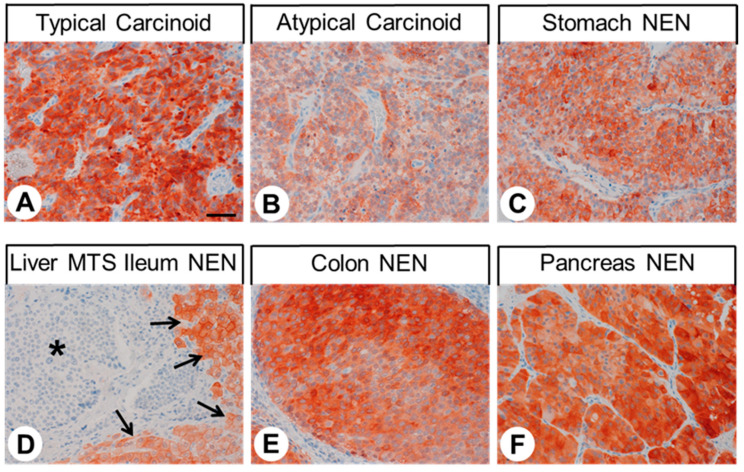
GPER expression pattern in different bronchopulmonary and gastroenteropancreatic neuroendocrine tumour entities. Immunohistochemical staining (red-brown colour) and counterstaining with haematoxylin. Scale bar: 100 µm (**A**–**F**). Asterisk in (**D**), liver metastasis (MTS) of an ileum neuroendocrine neoplasm (NEN); arrows in (**D**), surrounding GPER-positive non-neoplastic liver tissue.

**Figure 7:**
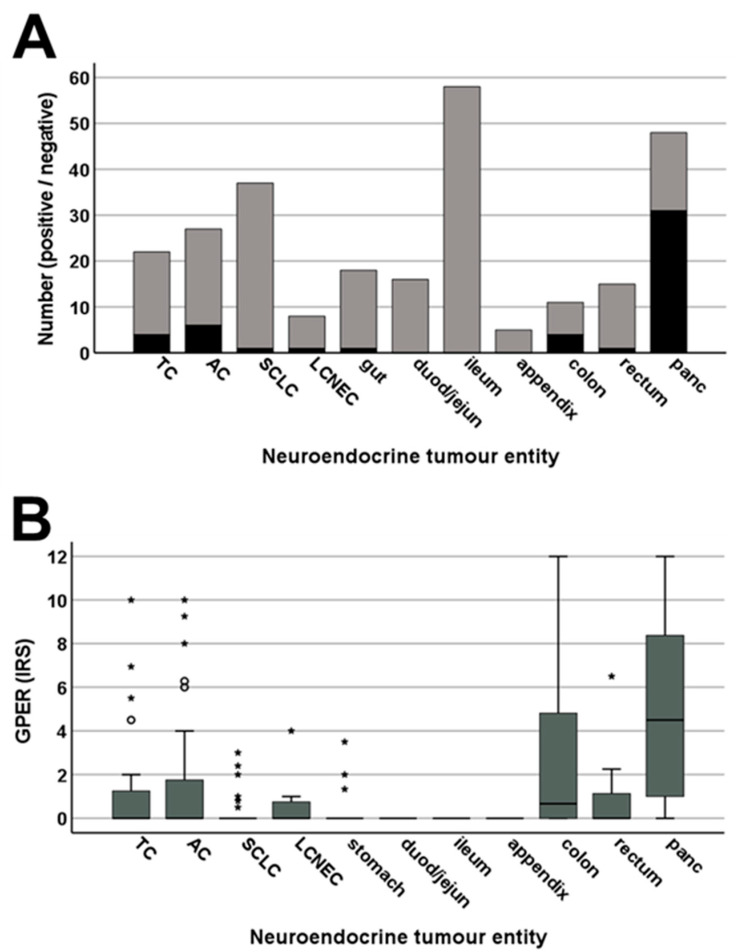
Expression profile of GPER in different bronchopulmonary and gastroenteropancreatic neuroendocrine tumour entities. (**A**) Number of GPER-positive (black) and GPER-negative cases (grey) within the different neuroendocrine tumour entities. Tumours were only considered positive at immunoreactivity score (IRS) values ≥ 3. (**B**) GPER expression levels (IRS values) in the different neuroendocrine tumour entities. Median values, upper and lower quartiles, minimum and maximum values, and outliers are shown. Outliers were defined as follows: circles, mild outliers (data points between 1.5 and 3 times above the upper quartile or below the lower quartile); asterisks, extreme outliers (data that fell more than 3 times above the upper quartile or below the lower quartile). TC, typical carcinoid of the lung; AC, atypical carcinoid of the lung; SCLC, small cell lung cancer; LC-NEC, large-cell neuroendocrine carcinoma of the lung; gut, gastroenteropancreatic neuroendocrine tumour (GEP-NEN) from the gut; duod/jejun, GEP-NEN from the duodenum or jejunum; ileum, GEP-NEN from the ileum; colon, GEP-NEN from the colon; rectum, GEP-NEN from the rectum; panc, pancreatic neuroendocrine tumour.

Figure 6 shows representative images of GPER immunostaining in different bronchopulmonary and gastroenteropancreatic neuroendocrine tumours. Distinct immunostaining of the cytoplasm and the plasma membrane of the tumour cells was observed. Overall, based on the percentage of GPER-positive tumours (IRS ≥ 3; Figure 7A) and the extent of GPER expression (Figure 7B), GPER was most prominently expressed in neuroendocrine tumours derived from the pancreas (64.6% GPER-positive cases; median IRS value, 4.5), followed by those derived from the colon (36.4% GPER-positive cases; median IRS value, 2.8) and the rectum (6.7% positive cases; median IRS value, 0.9), as well as atypical carcinoids (22.2% GPER-positive cases; median IRS value, 1.9) and typical carcinoids of the lung (18.2% GPER-positive cases; median IRS value, 1.4). However, expression levels varied considerably between individual patients and sometimes between different samples from the same patient, which is illustrated by the lengths of the respective boxes and whiskers in Figure 7B. For pancreatic tumours and tumours from the colon, IRS values ranged from 0 points (no expression) to 12 points (maximum expression).

#### 2.4.2. Correlations with Clinical Data

Due to known biological differences, e.g., regarding expression and prognostic significance of cytokeratins, transcription factors, peptide hormones or receptor markers such as somatostatin receptors (SST) [27,28,29], correlations between GPER expression and clinical data were calculated separately for BP-NEN and GEP-NEN.

When only the BP-NEN data were considered, no significant differences in GPER expression were noted, regardless of patient characteristics, including sex, age, smoking status, tumour size, or the presence of lymph node or distant metastases; however, a significant decline in GPER expression was observed as tumour grading increased (mean IRS values ± standard error of the mean (S.E.M.): G1 histology, 2.061 ± 0.599; G2 histology, 1.518 ± 0.701; G3 histology, 0.306 ± 0.118; Kruskal–Wallis test, *p* = 0.001). Correspondingly, a negative correlation between GPER expression and the expression of the proliferation marker Ki-67 (rsp = −0.369; *p* < 0.001) and a positive correlation between GPER expression and patient overall survival (rsp = 0.435; *p* < 0.001) were noted. Additionally, tumours from patients who were still living at the end of the observation period displayed significantly higher GPER IRS values than those who died from tumour-related causes (mean IRS values ± S.E.M.: alive, 1.386 ± 0.346; deceased, 0.518 ± 0.270; Mann–Whitney U test: *p* = 0.023). These findings were further corroborated by Kaplan–Meier and Cox regression analyses. When using either IRS ≥ 3 or IRS > 0 (the latter representing the overall median IRS value for BP-NEN) as the cut-off for GPER positivity, patients with GPER-positive tumours showed significantly better outcomes than those with GPER-negative tumours (log-rank test: cut-off IRS ≥ 3, *p* = 0.020; cut-off IRS > 0, *p* < 0.001). In the Cox regression analysis, a significant negative association between the GPER IRS values and time to tumour-related death was also observed (*p* = 0.047).

Interrelationships were also analysed between GPER expression and the expression of several typical neuroendocrine tumour markers, including chromogranin A (CgA), programmed death-ligand 1 (PD-L1), the chemokine receptor CXCR4, and the somatostatin receptors (SST) 1–5, which were determined on the same set of samples as part of previous studies [28,30,31]. A positive correlation was noted between GPER expression and expression of the markers known to be associated with a good prognosis for BP-NEN [27], including SST1 (rsp = 0.322; *p* = 0.002) and CgA (rsp = 0.287; *p* = 0.007), whereas no interrelationships were identified for any other investigated marker.

When only GEP-NEN were included in the analysis, again, no significant differences in GPER expression were noted, regardless of patient characteristics, including sex, age, tumour size, or the presence of regional or distant metastases. In contrast to BP-NEN, a positive association was found in GEP-NEN between GPER and Ki-67 expression (rsp = 0.281; *p* < 0.001) and between GPER expression and the expression of typical markers associated with a negative prognosis for cancer, including the chemokine receptor CXCR4 (rsp = 0.191; *p* = 0.013) and PD-L1 (rsp = 0.212; *p* = 0.006), whereas there was a negative correlation with CgA, which is associated with a good prognosis for GEP-NEN (rsp = −0.159; *p* = 0.040) [30,32]. In addition, significant positive interrelationships were observed between GPER and both SST1 (rsp = 0.370; *p* < 0.001) and SST5 expression (rsp = 0.202; *p* = 0.009). No significant influence was noted for GPER expression on patient overall survival, and no significant difference was observed between the GPER IRS values for patients who were still alive at the end of the observation period and those who had died.

Because GPER has been shown to be present in normal glucagon-, insulin-, and somatostatin-producing islet cells, pancreatic neuroendocrine tumours were also evaluated for glucagon, insulin, and somatostatin 14/28 expression. As expected, a strong positive relationship was found between the IRS values for GPER and those for glucagon (rsp = 0.382; *p* < 0.008), insulin (rsp = 0.462; *p* = 0.001), and somatostatin-14/28 (rsp = 0.312; *p* = 0.035).

## 3. Discussion

### 3.1. Characterisation of the Rabbit Monoclonal Anti-Human GPER Antibody 20H15L21

Monoclonal antibodies are advantageous over polyclonal ones because they are directed against a single epitope, which typically results in more specific staining. Additionally, and most importantly, they are available in unlimited quantities and with consistent quality. Because no such antibody was commercially available for GPER, we aimed to develop a monoclonal anti-GPER antibody that could be used for both basic research applications, including Western blot analyses and immunocytochemistry, and clinical applications, such as the immunohistochemical staining of formalin-fixed, paraffin-embedded tissues as performed during routine histopathology analyses. In the present study, we demonstrate that a rabbit monoclonal antibody raised against an epitope of the carboxyl-terminal tail of human GPER is effective for use in all three of the most common antibody applications, including immunofluorescent double-labelling of paraffin-embedded sections.

We present strong evidence that the novel anti-GPER antibody 20H15L21 specifically detects its targeted receptor and does not cross-react with other proteins. First, in Western blot analyses, the anti-GPER antibody selectively detected its cognate antigen in crude extracts obtained from GPER-transfected HEK-293 cells, native endogenous GPER-expressing A431 cells, and human liver, with bands appearing at the expected molecular weights of the differently glycosylated receptor and a proteolytically degraded form of GPER [26]. In contrast, no protein was detected in extracts from mock-transfected HEK-293 cells, which do not express endogenous GPER. In A431 cells, we additionally performed siRNA knockdown as well as preadsorption experiments. These studies revealed a reduction of the immunosignal after siRNA treatment of the cells, and a complete extinction of the bands after preincubation of the antibody with the immunising peptide but not with the peptide corresponding to another region of the receptor. However, in contrast to WGA bead-enriched samples from GPER-HEK-293 cells, non-enriched samples from A431 cells only contained the 40 kDa species of the receptor. The 40 kDa species was also the predominant form in non-enriched preparations from GPER-HEK-293 cells and human liver where the expression levels of the 55–70 kDa and 35 kDa species of the receptor were probably too low for detection by Western blot analysis without prior enrichment using WGA beads. According to data in the literature [26], the GPER matures only very slowly and remains linked to the endoplasmic reticulum as a 40 kDa species, whereas the 55–70 kDa species represents the mature form of the receptor that is transferred to the plasma membrane. The 35 kDa species, on the other hand, was suggested to represent a proteolytically degraded form. According to our findings, all three species (and, at least partly, the 40 kDa species) seem to represent glycosylated forms of the GPER since they all can be enriched by means of WGA beads.

Second, the antibody revealed cytoplasmic staining in both GPER-transfected HEK-293 cells and endogenous GPER-expressing A431 and BON-1 cells, and this staining could be completely extinguished by preadsorption of the antibody with the immunising peptide, but not with a peptide representing another region of the GPER receptor.

Third, the immunosignal observed in A431 and BON-1 cells was completely abolished by the treatment of these cells with a specific GPER-targeting siRNA.

Fourth, the novel antibody 20H15L21 yielded highly efficient staining in distinct cell populations known to express GPER [19] within formalin-fixed, paraffin-embedded human tissue samples. When comparing the novel monoclonal antibody with two commercially available polyclonal antibodies commonly used for immunohistochemical staining in the literature, similar results were obtained. With the novel monoclonal antibody, however, a more distinct immunosignal and less non-specific nuclear and background staining were observed. Furthermore, an interrelationship was noted between GPER protein levels as determined with the novel antibody and GPER mRNA expression levels as measured by qRT-PCR in serial sections of various tumours. Finally, the observed tissue immunostaining was completely abolished by preadsorption of the novel antibody with its immunising peptide but not with a peptide from another region of the GPER.

In the immunocytochemistry experiments, we observed a predominant intracellular localisation pattern for both transfected and endogenous GPER, which did not change substantially following stimulation with the selective GPER agonist G-1. This finding corresponds with data reported in the literature showing that the vast majority of GPER is localised on intracellular membranes, such as the endoplasmic reticulum, where it is also able to initiate signalling [33]. GPER trafficking appears to be complex, and cell surface receptors have been posited to undergo constitutive clathrin-mediated internalisation resulting in trafficking to intracellular membranes [15]. In the present study, however, after the cells were treated with the selective GPER antagonist G-15, the immunosignal clearly shifted to the plasma membrane. As mentioned above, GPER matures only slowly. It may be possible that at the high concentration of 10 µM used in the present investigation, G-15 also acts as a pharmacological chaperone [34,35,36,37] for GPER, either by fostering maturation and trafficking of the receptor from the endoplasmic reticulum/Golgi apparatus to the plasma membrane, promoting recycling of the receptor after internalisation instead of degradation, or stabilizing the receptor at the plasma membrane.

### 3.2. Immunohistochemical Detection of GPER Expression in Normal Human Tissues

In the present study, strong GPER immunostaining was observed in the pyramidal cells of the human cortex, which is consistent with published data for GPER-LacZ reporter mice, as well as with reported findings at both the mRNA and protein levels in mice and rats [19,38,39]. We were also able to identify GPER expression in a subpopulation of cells within the anterior human pituitary, which also aligns well with the available data from GPER-LacZ reporter mice [19]. Furthermore, in the present investigation, a strong GPER positivity was observed in the vast majority of pancreatic islet cells, which were identified as glucagon-, insulin-, and somatostatin-producing cells, based on both their anatomical localisation within the islets and positive double-labelling for GPER with glucagon, insulin, or somatostatin-14/28. These results were further corroborated by a positive correlation between GPER and glucagon, insulin, or somatostatin-14/28 expression in pancreatic neuroendocrine tumour tissue, which was observed during the present investigation. GPER expression in glucagon-, insulin-, and somatostatin-producing islet cells has also been demonstrated in mice [19,20,21], and GPER stimulation has been associated with an increase in insulin and a reduction in glucagon secretion [19,20,21]. GPER expression has also been detected in GPER-LacZ reporter mice in the gastric chief cells at the base of the fundic glands [19], which suggests the involvement of GPER in the regulation of pepsinogen secretion. In addition, we were able to demonstrate the presence of GPER in the epithelium and Brunner’s glands of the duodenum and in the epithelium of the gallbladder. At these sites, GPER may be involved in bicarbonate and mucin secretion. In the human liver, GPER expression has previously been described using polyclonal antibodies [40,41], and treatment with 17β-oestradiol increased human primary hepatocyte proliferation and accelerated hepatocarcinogenesis in zebrafish. This observed tumorigenic effect was blocked by the chemical inhibition or genetic knockout of GPER, indicating that the GPER might be involved in the regulation of liver growth during development, regeneration, and carcinogenesis [41]. GPER expression was also previously detected in the proximal tubules of the kidneys in rats [42]. Coexpression with megalin was also demonstrated, and a potential influence of GPER on megalin-induced protein absorption was postulated [38]. Furthermore, GPER has been detected in the adrenal medulla at the protein level in rats and mice [19,39] and at the mRNA level in humans [43]. Finally, a similar staining pattern for GPER in the human placenta as that detected in the present investigation was reported using polyclonal antibodies, and the authors of the previous study suggested that placental development is at least partially mediated via GPER [44].

In the present study, GPER expression was not detectable in the human thymus or other lymphatic organs, which corresponds well with the respective findings reported for GPER-LacZ mice [19] and with the observation that GPER-knockout mice display no gross immunological defects [19]. GPER was also absent in the human heart, which contrasts with reports in the literature that cardiomyocyte-specific GPER deletion in mice leads to left ventricular dysfunction and adverse remodelling [45], suggesting some species-specific differences in GPER expression in this respect. No GPER expression was detected in normal prostate, testicular, breast, ovarian, or uterine tissue in the present study, which agrees with the finding that GPER-knockout mice, unlike ERα or ERβ knockout animals, do not show impairment of fertility or changes in the morphology of the reproductive organs. The selective GPER agonist G-1, despite affecting glucose homeostasis in mice, does not appear to influence the uterus [19,20,22,23,24,25,46]. However, using polyclonal antibodies, GPER protein was detected in the testicular peritubular cells and vessels in rhesus monkeys, and decreased receptor expression was associated with impaired spermatogenesis [47]. Therefore, some species differences in GPER expression in the reproductive organs may exist. Using polyclonal antibodies in biopsies from the tibial and femoral growth plates of children, GPER expression has been detected in osteocytes, osteoblasts, and osteoclasts; however, there was a steep decline in receptor expression after puberty [48]. In our bone specimens, which were derived from the femoral heads of patients aged between 75 and 82 years who underwent hip replacement therapy, no GPER expression was detectable. These findings suggest that GPER may only be present in the growth plates of juveniles and is not expressed in aged bone.

### 3.3. Immunohistochemical Detection of GPER Expression in Human Neoplastic Tissues

Among the 31 tumour entities investigated in the present study, notable GPER expression (mean IRS value ≥ 3) was only observed in hepatocellular, pancreatic, and renal clear cell carcinomas, pancreatic neuroendocrine tumours, pheochromocytomas, and endometrial cancer. Among these few tumour entities, moderate to strong, and potentially clinically relevant GPER expression (mean IRS ≥ 6), was only detected in pancreatic and renal clear cell carcinomas.

In the literature, studies have primarily been performed on sex hormone-dependent tumours, with contradictory results regarding the extent of GPER expression relative to non-neoplastic tissues and the impact on patient outcomes, both among different tumour entities and within a given tumour type. The divergent results reported by the various immunohistochemical investigations may in part be due to the wide variety of polyclonal antibodies used across studies, which were derived from different commercial and non-commercial sources; however, some differences may also be associated with variations in the signalling pathways utilised by GPER in different tumour types (and potentially in different tumour subtypes) [1,15]. Available data concerning non-sex hormone-dependent tumour entities are much less extensive but are often also divergent. To the best of our knowledge, no GPER expression data are available for glioblastomas, medullary and anaplastic thyroid carcinomas, parathyroid adenomas, gastrointestinal stromal tumours, cholangiocellular carcinomas, BP-NENs or GEP-NENs (apart from SCLC), renal cancer, pheochromocytomas, lymphomas, melanomas, or sarcomas.

In the literature, a high level of GPER expression has been reported in all types of **lung cancer**, whereas the surrounding normal lung tissue was largely negative [49,50]. However, in in vitro studies on non-SCLC (NSCLC) cell lines both decreased [51] and increased [50] cell migration and invasion capacities after GPER stimulation have been observed. These results correspond with our findings regarding non-neoplastic lung tissue; however, in our study, only individual cases of SCLC and NSCLC were GPER-positive.

In papillary and follicular **thyroid cancer,** an upregulation of GPER expression in comparison to normal thyroid tissue has been observed [52,53]. Additionally, in papillary tumours, high GPER expression was associated with the presence of lymph node metastases [52], and in thyroid cancer cell lines, stimulation of GPER increased cell proliferation, migration, and invasion [12,54].

A steep decline to practically no GPER expression in comparison with normal tissue, and a further decline associated with advanced stages, have been shown in the literature for **gastric cancer** [55,56] and **colon cancer** [57]. In gastric cancer, the decrease in GPER expression was also associated with poor patient outcomes [56]. Experiments performed in gastric cancer cell lines also indicated that GPER might act as a tumour suppressor [55]. Overall, these findings fit well with the results of the present investigation showing the presence of GPER only in individual cases.

In **hepatocellular carcinomas** (HCC), reports describing significantly higher GPER expression in malignant tissue than normal liver tissue [41] contrast with studies showing significantly reduced GPER expression at both the mRNA and protein levels compared with the surrounding non-malignant tissue [58,59]. In the present investigation, GPER expression levels were distinctly lower in the investigated HCC samples (mean IRS, 3.09) than in the surrounding non-malignant tissue (mean IRS, 5.21). GPER appears to act as a tumour suppressor in HCC, since GPER-knockout mice displayed increased tumorigenesis in a diethylnitrosamine-induced liver tumour model [58] and stimulation of GPER reduced HCC xenograft growth in mice [59].

In the literature, presence of GPER has been demonstrated in the majority of **pancreatic adenocarcinoma** samples [60], which fits well with the results of the present investigation. Additionally, the proliferation of pancreatic cancer cell lines and pancreatic cancer xenografts in mice was significantly reduced following GPER stimulation [60].

A decline in GPER expression with increasing malignancy has been observed in **prostate** adenocarcinomas [61], and cell growth was inhibited in PC-3 cells following GPER stimulation [62].

By contrast, significant GPER expression has been described in **testicular** germ and Leydig cell tumours [63,64,65]; however, unlike lung, gastric, colon, and HCC cancer cell lines, GPER stimulation caused an increase in the proliferation of JKT-1 seminoma cells [65].

Data in the literature regarding the presence of GPER in **breast cancer** are contradictory. In some studies, GPER expression has been shown to decrease with histological grading and the presence of lymph node metastases, and this decline in expression was associated with poor patient outcomes [66,67,68,69]. Other investigations, by contrast, have revealed an overexpression of GPER in breast cancer, a positive correlation between GPER expression and tumour size, and a negative correlation between GPER expression and prognosis [70,71,72,73,74]. Data regarding the influence of GPER on breast cancer cell lines are also inconsistent. Some studies reported inhibition of cell proliferation after GPER stimulation [75,76,77,78,79], but others, including some studies performed with the same cell lines, have reported a GPER-mediated stimulation of cell growth [71,80,81].

GPER expression in **ovarian cancer** is also controversial. Some studies have shown higher GPER expression in benign tissue and low-malignancy tumours than in ovarian cancer and reported that GPER was predominantly detected during the early stages of the disease and in tumours with low grading, with high GPER expression associated with favourable clinical outcomes [82,83,84]. In addition, a GPER-mediated suppression of cell proliferation has been demonstrated in SK-OV-3, OVCAR-3, and OAW-42 ovarian cancer cell lines [82,83,84]. However, increased GPER expression in ovarian cancer relative to tumours with low malignant potential has also been noted, and the elevated GPER expression in these studies was linked to poor survival [85,86]. Correspondingly, in the ovarian cancer cell lines BG-1, 2008, and OVCAR-5, GPER mediated an increase in cell proliferation [80,87]. In another study, no differences in GPER expression between benign and malignant tumours, and no correlations between GPER staining and clinical stage, histological grade, or patient survival were noted [88].

Likewise, in **endometrial cancer** reports on a decreased GPER expression relative to normal tissue and on a GPER-mediated inhibition of tumour cell proliferation [89,90] contrast with other studies showing an increased presence of the receptor in tumours, a rise in expression with increased grading and staging, poor survival among patients with high GPER expression, and an increase in the growth of endometrial cancer cell lines after GPER stimulation [91,92,93,94].

Finally, in **cervical cancer**, increased GPER expression compared with non-neoplastic tissue was noted along with an association between high GPER expression and poor patient outcomes [95].

Aside from endometrial cancer, the present investigation did not detect notable GPER expression in sex hormone-dependent tumours, although the receptor was detected in some individual cases. Thus, our findings align with those studies that show a decrease in GPER expression in malignant tissues. However, since we have investigated only a limited number of cases per tumour entity, further investigation with larger case numbers per tumour type are necessary in order to adequately survey the rarer tumour subtypes (with possibly different GPER expression levels). Here, the novel antibody 20H15L21 may serve as a valuable tool.

Among the large panel of neuroendocrine tumours investigated, distinct differences in GPER expression were observed depending on the localisation of the tumour entities, with the highest proportion of positive cases and the highest average IRS values observed for tumours of the pancreas compared with tumours from other sites. This outcome was expected because among the respective non-neoplastic tissues, GPER was only notably detected in pancreatic islets, and not in the neuroendocrine cells of the bronchopulmonary or gastrointestinal tract. In BP-NEN, a decline in GPER expression was observed with increased grading, a negative correlation was identified with the Ki-67 index, and positive correlations were observed with patient outcomes and markers associated with a good prognosis, indicating that GPER acts as a tumour suppressor and may also serve as prognostic marker. In GEP-NEN, by contrast, positive correlations were found for GPER expression with the Ki-67 index and markers associated with poor prognosis, and negative associations were identified for markers predicting favourable patient outcomes, although no significant impact on overall patient survival was observed. Therefore, the present study confirms reports in the literature describing the different biology between BP-NEN and GEP-NEN [27,28,29]. Our data further corroborate findings from the literature that indicate that the effects of GPER on tumour cell growth may vary depending on the tumour type or subtype. Although GPER expression may serve as a prognostic marker in BP-NEN, the overall expression levels of GPER in neuroendocrine tumours are generally too low to be of clinical relevance, except in individual cases, particularly for pancreatic neoplasms.

## 4. Materials and Methods

### 4.1. Antibody

A rabbit monoclonal antibody, 20H15L21, was generated against the carboxyl-terminal tail of human GPER in collaboration with and obtained from Thermo Fisher Scientific (Waltham, MA, USA). The identity of the peptide used for immunisation of rabbits was HAALKAVIPDSTEQSDVRFSSAV, corresponding to residues 353–375 of human GPER. This sequence is unique for human GPER, and because of dissimilarities in the carboxyl-terminal amino acid sequence, 20H15L21 does not cross-react with rat or mouse GPER.

### 4.2. Western Blot Analysis

Human embryonic kidney 293 (HEK-293) cells (DMSZ, Braunschweig, Germany) were either mock-transfected or transfected with a plasmid encoding a HA-tagged human GPER (GPER-HEK-293 cells) using Lipofectamine 2000 according to the instructions of the manufacturer (Invitrogen, Carlsbad, CA, USA). Stable transfectants were selected in the presence of 400 µg/mL G418. GPER-HEK-293 cells or endogenous GPER-expressing BON-1 or A431 cells (DMSZ, Braunschweig, Germany) were seeded onto poly-L-lysine-coated 60-mm dishes and grown to 80% confluency. If indicated, GPER expression was silenced in A431 cells using chemically synthesised, double-stranded GPER small interfering RNA (siRNA) duplexes (sc-60743; Santa Cruz Biotechnology, Dallas, TX, USA) according to the manufacturer’s instructions. As a negative control, an empty vector was used (Santa Cruz Biotechnology, Dallas, TX, USA). Cells were lysed in detergent buffer (20 mM HEPES (pH 7.4), 150 mM NaCl, 5 mM EDTA, 1% Triton X-100, 10% glycerol, 0.1% SDS, 0.2 mM phenylmethylsulfonylfluoride, 10 mg/mL leupeptin, 1 mg/mL pepstatin A, 1 mg/mL aprotinin, and 10 mg/mL bacitracin). For the human liver samples, 50 mg of tissue was weighed, 200 µL of the detergent buffer was added, and the samples were sonicated for 10 s. Afterwards, the samples were gently inverted for 1 h at 4 °C before centrifugation for 30 min at 14,800× *g* at 4 °C. If indicated, GPER was enriched using wheat germ lectin agarose (WGA) beads (J-OIL MILLS Inc., Tokyo, Japan) as described previously [96]. Samples were then subjected to 7.5% sodium dodecyl sulfate-polyacrylamide gel electrophoresis and immunoblotted onto polyvinylidene fluoride membranes. Blots were incubated with rabbit monoclonal anti-GPER antibody 20H15L21 at a dilution of 1:500 overnight at 4 °C, followed by incubation with 1:5000-diluted peroxidase-conjugated secondary anti-rabbit antibody (Santa Cruz Biotechnology, Dallas, TX, USA) for 2 h at room temperature and visualised with enhanced chemiluminescence (Amersham, Braunschweig, Germany). For adsorption controls, the anti-GPER antibody was preincubated for 2 h with 10 µg/mL of the peptide used for rabbit immunisations (peptide 1) or 10 µg/mL of a peptide representing the amino acid sequence at the N-terminal region of the receptor (peptide 2, DVTSQARGVGLEMYPGTA, corresponding to amino acids 2–19 of GPER).

### 4.3. Immunocytochemistry

GPER-HEK-293 cells or endogenous GPER-expressing BON-1 or A431 cells were grown on coverslips overnight and either left untreated or exposed to 10 µM selective GPER agonist G-1 or 10 µM selective GPER antagonist G-15 (Tocris, Bristol, UK) for 30 min for a clear-cut effect. According to data in the literature, G-1 and G-15 at this high concentration display little or no binding to ERα or ERβ [16,17,80]. In an additional set of experiments, GPER expression was silenced using chemically synthesised, double-stranded GPER small interfering RNA (siRNA) duplexes (sc-60743; Santa Cruz Biotechnology, Dallas, TX, USA) according to the manufacturer’s instructions. As a negative control, an empty vector was used (Santa Cruz Biotechnology, Dallas, TX, USA). Cells were then fixed with 4% paraformaldehyde and 0.2% picric acid in phosphate buffer (pH 6.9) for 20 min at room temperature, washed with phosphate buffer, and incubated with anti-GPER antibody 20H15L21 (1:500) overnight at 4 °C, followed by an Alexa Fluor 488-conjugated secondary antibody (Invitrogen, Carlsbad, CA, USA; dilution: 1:5000) for 2 h at room temperature. Samples were mounted with Fluoromount G (Invitrogen, Carlsbad, CA, USA) and examined using a Zeiss LSM 510 META laser-scanning confocal microscope (Jena, Germany). For immunostaining controls, the anti-GPER antibody was either omitted or preincubated for 2 h with 10 µg/mL of the peptide used for rabbit immunisations (peptide 1) or 10 µg/mL of a peptide representing the amino acid sequence at the N-terminal region of the receptor (peptide 2, DVTSQARGVGLEMYPGTA, corresponding to amino acids 2–19 of GPER).

### 4.4. Immunohistochemical Evaluation of GPER Expression in Normal and Neoplastic Human Tissues

Tissue Specimens

For the initial evaluation of GPER expression in different human tumour entities, 273 formalin-fixed, paraffin-embedded tumour samples (neuroendocrine tumours, originally 10 samples; Table 1) were obtained from the Department of Pathology of the Ernst-Moritz-Arndt-University (Greifswald, Germany) and the Laboratory of Pathology and Cytology Bad Berka (Bad Berka, Germany). Many of the tumour specimens contained adjacent non-malignant tissues, which were also analysed. Additionally, tumour-free human tissue samples from the cortex, pituitary, lung, heart, liver, gallbladder, stomach, gut, pancreas, kidney, spleen, tonsils, thymus, lymph nodes, testicles, placenta, and bone (*n* = 5–24 each) were obtained from the Department of Pathology of the Ernst-Moritz-Arndt-University (Greifswald, Germany) and the Laboratory of Pathology and Cytology Bad Berka (Bad Berka, Germany) for evaluation. Staining patterns in malignant tissues were compared with those observed in the non-malignant tissues surrounding the tumours.

### 4.5. Bronchopulmonary and Gastroenteropancreatic Neuroendocrine Neoplasms

#### 4.5.1. Tumour Samples

For the subsequent assessment of GPER expression BP-NENs and GEP-NENs, a total of 768 tumour samples from 282 patients were evaluated (in detail, 114 patients with only 1 sample, 55 patients with 2 samples, 44 patients with 3 samples, 32 patients with 4 samples, 13 patients with 5 samples, 8 patients with 6 samples, 3 patients with 7 samples, 3 patients with 8 samples, 3 patients with 9 samples, 1 patient with 11 samples, 1 patient with 12 samples, 3 patients with 14 samples, 1 patient with 16 samples, and 1 patient with 18 samples; 415 were primary tumour samples, 315 were metastatic samples, and for 38 samples, this information was not included in the patient records). From some patients, both primary and metastatic samples were available. Of the 282 tumours, 94 (33.3%) originated from the lungs (in detail, 22 typical carcinoids (TC), 27 atypical carcinoids (AC), 37 small cell lung cancer (SCLC), and 8 large-cell neuroendocrine carcinomas (LCNEC), 18 (6.4%) from the stomach, 16 (5.7%) from the duodenum/jejunum, 58 (20.6%) from the ileum, 5 (1.8%) from the appendix, 11 (3.9%) from the colon, 15 (5.3%) from the rectum, and 48 (17.0%) from the pancreas. The localisation of 17 (6.0%) primary tumours was unknown. The samples were provided by the Institute of Pathology and Cytology Bad Berka (Bad Berka, Germany) and were surgically removed between 1998 and 2016 at the Department of General and Visceral Surgery, Zentralklinik Bad Berka (Bad Berka, Germany). Clinical data were gathered from patient records.

Permission was obtained from the local ethics committee (Ethikkommission der Landesärztekammer Thüringen) for this retrospective analysis. All data were recorded and analysed anonymously.

#### 4.5.2. Patient Characteristics

The neuroendocrine tumours evaluated in the present investigation were obtained from 142 men (50% of cases) and 123 women (44%). The sex of 17 patients (6%) was unknown. The overall mean age of the patients at diagnosis was 58.7 years (median, 59.7 years; range, 12.1–83.9 years). Forty-six of the (corresponding) primary tumours (16.3%) were classified as T1, 46 (16.3%) as T2, 57 (20.2%) as T3, and 24 (8.5%) as T4. In 109 cases (38.7%), the extent of the primary tumour was not reported. Lymph node metastases were disclosed in 123 cases (43.6%), whereas 82 patients (29.1%) had none. For 77 patients (27.3%), the lymph node status was not known. Distant metastases were already present in 113 patients (40.1%), whereas 88 patients (31.2%) had no distant metastases, and the presence of distant metastases was not reported for 81 patients (28.7%). At diagnosis, 18 of 171 patients (10.5%) with GEP-NEN had Union for International Cancer Control stage I disease, 12 patients (7.0%) had stage II disease, 24 patients (14.0%) had stage III disease, and 101 patients (59.1%) had stage IV disease. For 16 GEP-NEN patients (9.4%), the disease stage was unknown. The disease stage was not determined for the 91 BP-NEN patients or the 20 patients with unknown tumour origins. Histological assessment showed that 106 patients (37.6%) had grade 1 tumours, 86 patients (30.5%) had grade 2 tumours, and 73 patients (25.9%) displayed grade 3 tumours. Tumour grading was not reported for 17 (6.0%) patients. The median overall follow-up time was 52.6 months. At the end of the follow-up period, 155 patients were still living, and 86 patients had died. For 41 patients, no survival data were available. Among the patients who died, the median survival time was 27.9 months.

### 4.6. Immunohistochemistry

From the paraffin blocks, 4-µm sections were prepared and floated onto positively charged slides. Immunostaining was performed using an indirect peroxidase labelling method, as described previously [97]. Briefly, sections were dewaxed, microwaved in 10 mM citric acid (pH 6.0) for 16 min at 600 W, and incubated with 20H15L21 (1:500) overnight at 4 °C. For comparison, a subset of serial sections was additionally incubated with rabbit polyclonal anti-GPER antibody ab39742, directed against the C-terminal tail of GPER (1:100; Abcam, Cambridge, UK), or with goat polyclonal anti-GPER antibody AF5534, directed against the N-terminal tail of GPER (1:200; R&D Systems, Minneapolis, MN, USA). Detection of the primary antibodies was performed using biotinylated anti-rabbit or anti-goat IgG, followed by incubation with peroxidase-conjugated avidin (Vector ABC “Elite” kit; Vector Laboratories, Burlingame, CA, USA). The binding of the primary antibodies was visualised using 3-amino-9-ethylcarbazole in acetate buffer (BioGenex, San Ramon, CA, USA). Sections were rinsed, counterstained with Mayer’s haematoxylin, and mounted in Vectamount™ mounting medium (Vector Laboratories, Burlingame, CA, USA). For immunohistochemical controls, 20H15L21 was either omitted or adsorbed for 2 h at room temperature with 10 µg/mL of peptide 1, which was used to immunise rabbits, or with peptide 2, which represents another region of the receptor (see “Western blot analysis”).

For dual immunofluorescence, sections were incubated overnight at 4 °C with 20H15L21 together with a mouse monoclonal anti-insulin antibody (1:100; Abcam, Cambridge, MA, USA), a mouse monoclonal anti-glucagon antibody (1:500; Sigma-Aldrich, St. Louis, MO, USA), or a rat anti-somatostatin-14/28 antibody (1:300; Abcam, Cambridge, MA, USA). Sections were incubated with Cy3-conjugated anti-rabbit and Alexa Fluor 488-conjugated anti-mouse or anti-rat antibodies. Specimens were mounted and examined using a Zeiss LSM 510 META laser-scanning confocal microscope.

GPER staining in tumour tissues was scored using the semiquantitative immunoreactivity score (IRS), as described by Remmele and Stegner (1987) [98]. The percentage of positive tumour cells quantified in five gradations (0, no positive cells; 1, <10% positive cells; 2, 10–50% positive cells; 3, 51–80% positive cells; 4, >80% positive cells) was multiplied by the staining intensity quantified in four gradations (0, no staining; 1, mild staining; 2, moderate staining; 3, strong staining). Thus, IRS values ranging from 0 to 12 were obtained. For neuroendocrine tumours, several patients had more than one tumour tissue sample. For these cases, an arithmetic mean was calculated from the IRS values of the different slides belonging to the same patient. Only tumours with an average IRS ≥ 3 were considered to be GPER-positive. All immunohistochemical staining was evaluated by two independent, blinded investigators (MB, AL). For discrepant scores, final decisions were achieved by consensus.

### 4.7. Quantitative Real-Time Polymerase Chain Reaction (qRT-PCR)

Adjacent paraffin sections from the immunohistochemistry slides from a series of selected tumour samples were analysed for GPER mRNA expression. The sections were purified and mRNA was isolated using a standardised isolation method based on magnetic beads (Extraction-XL (96) RNA 2.0 kit; STRATIFYER Molecular Pathology, Cologne, Germany) as previously described [99]. Directly after extraction, fully automated multiplex TaqMan-real-time PCR was performed using a SuperScript III Platinum One-Step real-time RT-PCR kit and Platinum Taq DNA polymerase (Invitrogen, Karlsruhe, Germany). Primers and probes for GPER mRNA variant 2, 3, and 4 were designed by STRATIFYER Molecular Pathology and prepared by Eurogentec (Seraing, Belgium). The housekeeping gene CALM2 (calmodulin 2) was used as a control. Additionally, a NTC (no-template control) and a human reference RNA (Agilent Technologies, Böblingen, Germany) were analysed in parallel. Measurements were conducted on an Mx3005P device using MxPro version 4.10d software (Agilent Technologies, Böblingen, Germany). After 40 cycles (50 min at 30 °C, 2 min at 95 °C, 40 × (15 s at 95 °C, 45 s at 60 °C)), a logarithmic analysis was performed with a threshold of 50. The values were normalised using the following formula: dCt(Norm) = 40 − ΔCt(Ct(GPER) − Ct(CALM2)). As a result, dCt values ≥ 19.00 were obtained and used for further calculations.

### 4.8. Statistical Analysis

For statistical analysis, SPSS 27.0.0.0 (IBM, Armonk, NY, USA) was used. Because the data were not normally distributed (Kolmogorov–Smirnov test), Kruskal–Wallis test, Mann–Whitney U test, Chi-square test, Kendall’s τ-b test, and Spearman’s rank correlation were performed. For survival analysis, the Kaplan–Meier method with a log-rank test and a Cox regression analysis were used. For all analyses, *p* values ≤ 0.05 were considered significant.

## 5. Conclusions

We have generated and characterised a novel rabbit monoclonal anti-human GPER antibody that is well-suited for basic research applications, including Western blot and immunocytochemical analyses, and for visualising human GPER in formalin-fixed, paraffin-embedded tissues during routine histopathological examinations.

This antibody enabled the detection of GPER protein expression in a wide variety of human normal and neoplastic tissues, some of which have not been evaluated for GPER expression so far, and, as a result, a broad expression profile for the human body and for human tumours could be established. However, with the exception of pancreatic and renal clear cell carcinomas and individual cases of pancreatic neuroendocrine neoplasms, our results indicate that GPER expression levels are too low in the vast majority of tumours to serve as a suitable diagnostic or therapeutic target. In BP-NEN, GPER expression may serve as positive prognostic marker.

## Figures and Tables

**Table 1:** Presence of GPER in different tumour entities. Tumours with mean IRS values ≥ 3.0 are marked in bold.

Tumour Type (Total Number of Cases)	GPER-Positive Tumours (*n*)	Immunoreactivity Score (IRS)		
		Mean	Min	Max
Glioblastoma (9)	0	0	0	0
Thyroid carcinoma (38)	0	0.11	0	2
-papillary (10)	0	0	0	0
-follicular (10)	0	0	0	0
-medullary (9)	0	0.22	0	2
-anaplastic (9)	0	0.22	0	2
Parathyroid adenoma (10)	2	2.00	0	9
Lung cancer (20)	3	1.08	0	8
-Adenocarcinoma (10)	1	0.8	0	8
-Squamous cell carcinoma (10)	2	1.35	0	4.5
Gastric adenocarcinoma (14)	0	0	0	0
Colon carcinoma (9)	2	1.33	0	6
Gastrointestinal stromal tumour (13)	0	0	0	0
**Hepatocellular carcinoma (11)**	5	**3.09**	0	12
Cholangiocellular carcinoma (9)	3	1.89	0	6
**Pancreatic adenocarcinoma (10)**	8	**6.45**	1	10
**Renal clear cell carcinoma (9)**	9	**8.89**	3	12
**Pheochromocytoma (7)**	3	**3.93**	0	8
Neuroendocrine tumour (273)	50	1.43	0	12
Prostate adenocarcinoma (24)	4	0.83	0	6
Testicular cancer (12)	0	0	0	0
Breast carcinoma (10)	0	0	0	0
**Endometrial cancer (10)**	9	**5.45**	2	8
Cervical cancer (9)	0	0.78	0	2
Ovarian cancer (10)	4	2.8	0	7.5
Lymphoma (10)	0	0	0	0
Melanoma (5)	0	0.40	0	2
Sarcoma (14)	0	0	0	0
-Liposarcoma (4)	0	0	0	0
-Rhabdomyosarcoma (3)	0	0	0	0
-Leiomyosarcoma (3)	0	0	0	0
-Pleomorphic sarcoma (2)	0	0	0	0
-Osteosarcoma (1)	0	0	0	0
-Ewing’s sarcoma (1)	0	0	0	0

## Data Availability

The data that support the findings of this study are all contained within the article.

## Supplementary Materials

The following are available online at https://www.mdpi.com/article/10.3390/ijms23095191/s1.

## Abbreviations

BP-NEN	bronchopulmonary neuroendocrine neoplasm
GEP-NEN	gastroenteropancreatic neuroendocrine neoplasm
GPR	G protein-coupled receptor
GPER	G protein-coupled oestrogen receptor
HEK	human embryonic kidney
IRS	immunoreactivity score
WGA beads	wheat germ lectin agarose beads
