# Supplementary material for: Assessment of G Protein-Coupled Oestrogen Receptor Expression in Normal and Neoplastic Human Tissues Using a Novel Rabbit Monoclonal Antibody
Source: Int J Mol Sci. 2022 May 6;23(9):5191. doi: 10.3390/ijms23095191 (PMC9099907; doi:10.3390/ijms23095191)
Supplement: Supplementary file 1 [file ijms-23-05191-s001.zip › Supplementary material_Bubb et al.pdf]

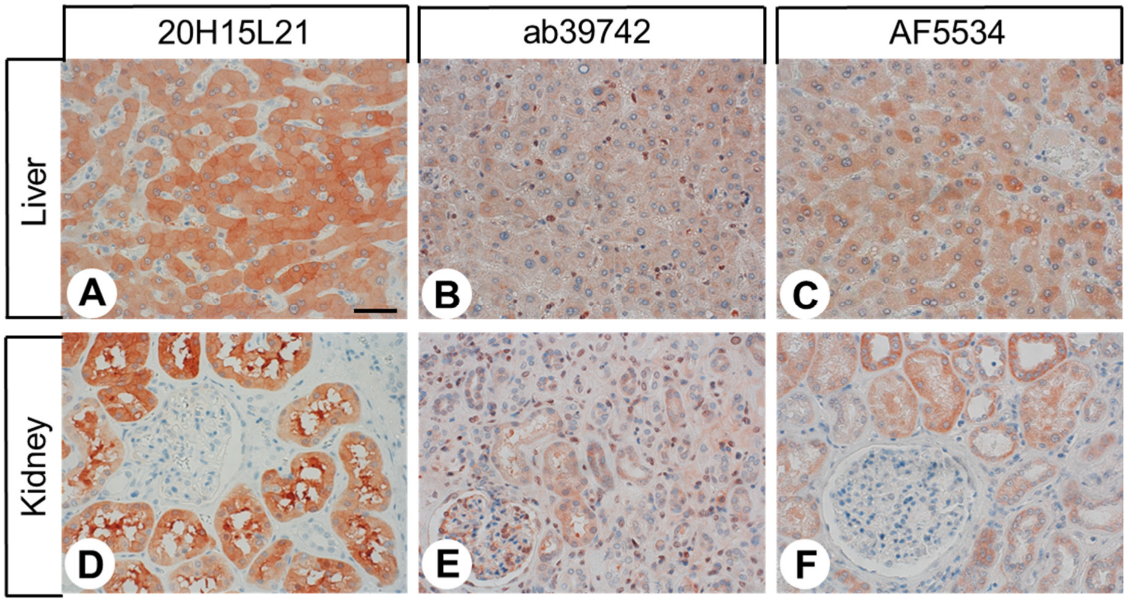


**Supplementary Figure S1:** Comparative immunohistochemical stainings of non-neoplastic human liver and kidney tissues with the novel monoclonal rabbit anti-GPER antibody 20H15L21 or with the rabbit polyclonal anti-GPER antibody ab39742 (Abcam) or with the goat polyclonal anti GPER antibody AF5534 (R&D Systems). Immunohistochemical staining (red-brown colour), counterstaining with haematoxylin. Scale bar: 100 µm (**A**–**F**).

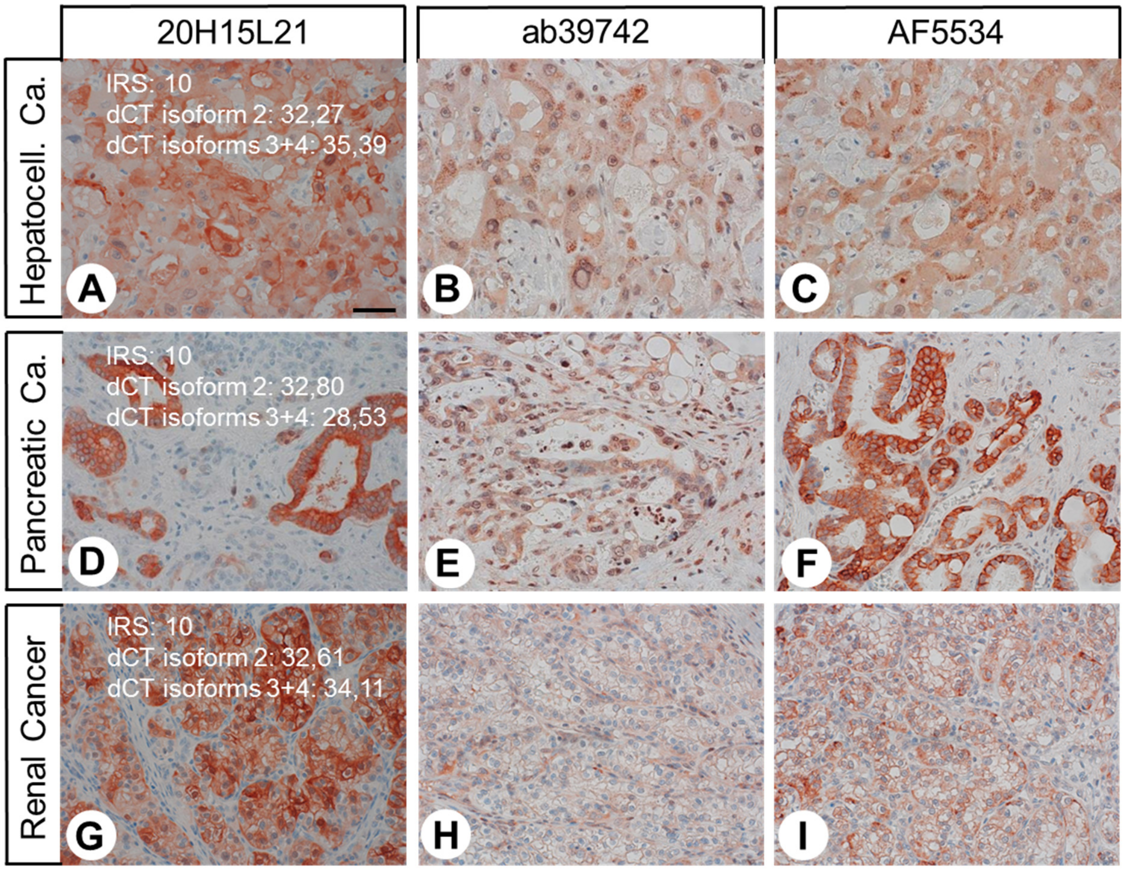


**Supplementary Figure S2:** Comparative immunohistochemical stainings of human neoplastic tissues with the novel monoclonal rabbit anti-GPER antibody 20H15L21 or with the rabbit polyclonal anti-GPER antibody ab39742 (Abcam) or with the goat polyclonal anti-GPER antibody AF5534 (R&D Systems). Immunohistochemical staining (red-brown colour), counterstaining with haematoxylin. Scale bar: 100 µm (**A**–**F**). **A, D, G**: IRS values obtained with the novel monoclonal rabbit anti-GPER antibody 20H15L21 in the respective tissue samples and dCT values obtained for the GPER mRNA variants 2 and 3+4 from serial sections by qRT-PCR.

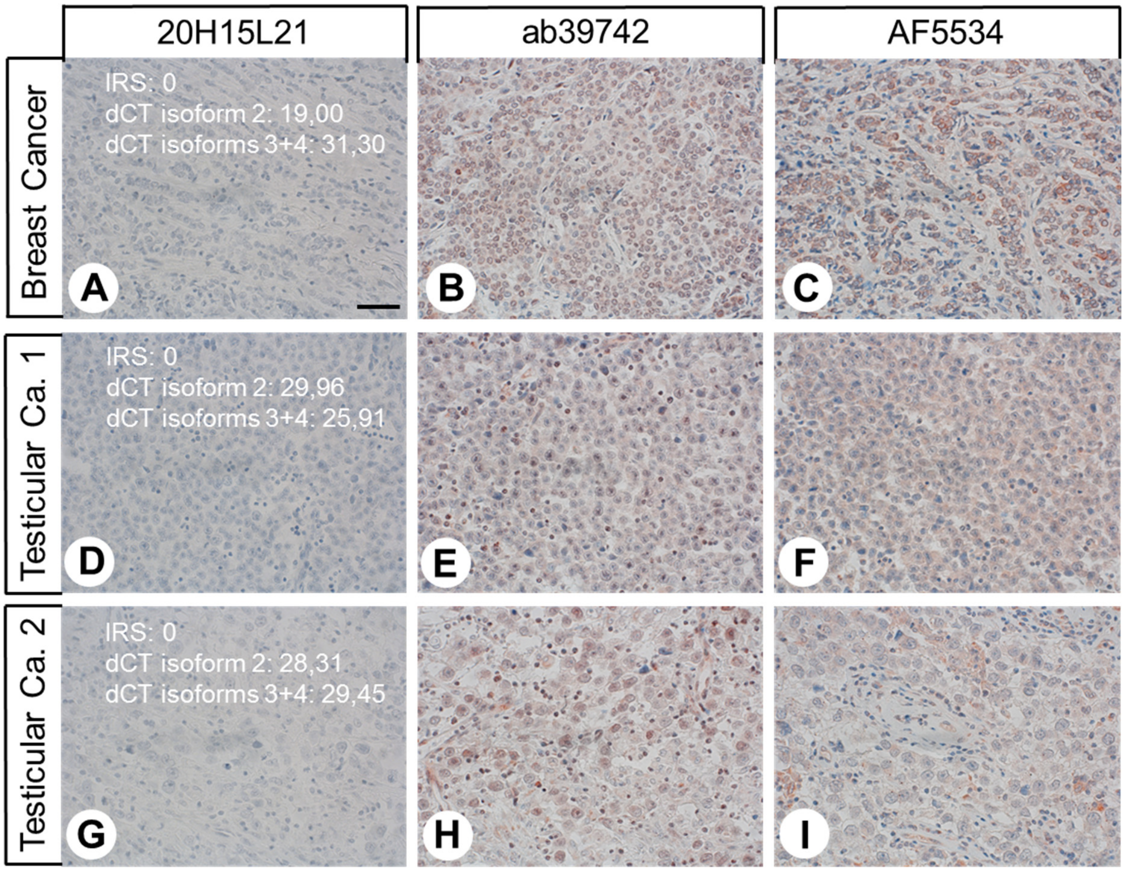


**Supplementary Figure S3:** Comparative immunohistochemical stainings of human neoplastic tissues with the novel monoclonal rabbit anti-GPER antibody 20H15L21 or with the rabbit polyclonal anti-GPER antibody ab39742 (Abcam) or with the goat polyclonal anti-GPER antibody AF5534 (R&D Systems). Immunohistochemical staining (red-brown colour), counterstaining with haematoxylin. Scale bar: 100 µm (**A**–**F**). **A, D, G**: IRS values obtained with the novel monoclonal rabbit anti-GPER antibody 20H15L21 in the respective tissue samples and dCT values obtained for the GPER mRNA variants 2 and 3+4 from serial sections by qRT-PCR.
