# Supplementary figures and images for: Assessment of G Protein-Coupled Oestrogen Receptor Expression in Normal and Neoplastic Human Tissues Using a Novel Rabbit Monoclonal Antibody
Source: Int J Mol Sci. 2022 May 6;23(9):5191. doi: 10.3390/ijms23095191 (PMC9099907; doi:10.3390/ijms23095191)

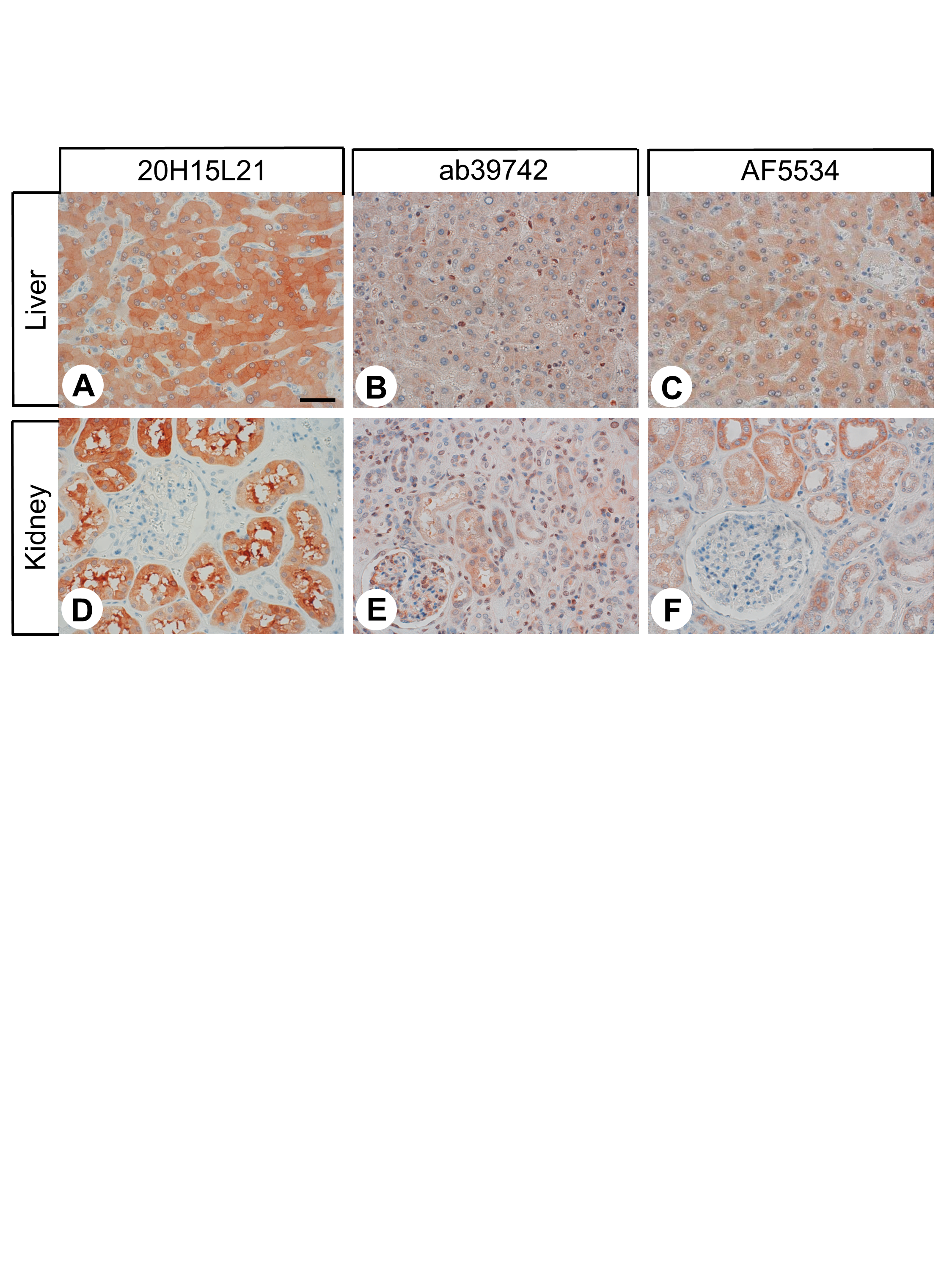

Supplement: Supplementary file 1 [file ijms-23-05191-s001.zip › Supplementary Figure 1.tif]

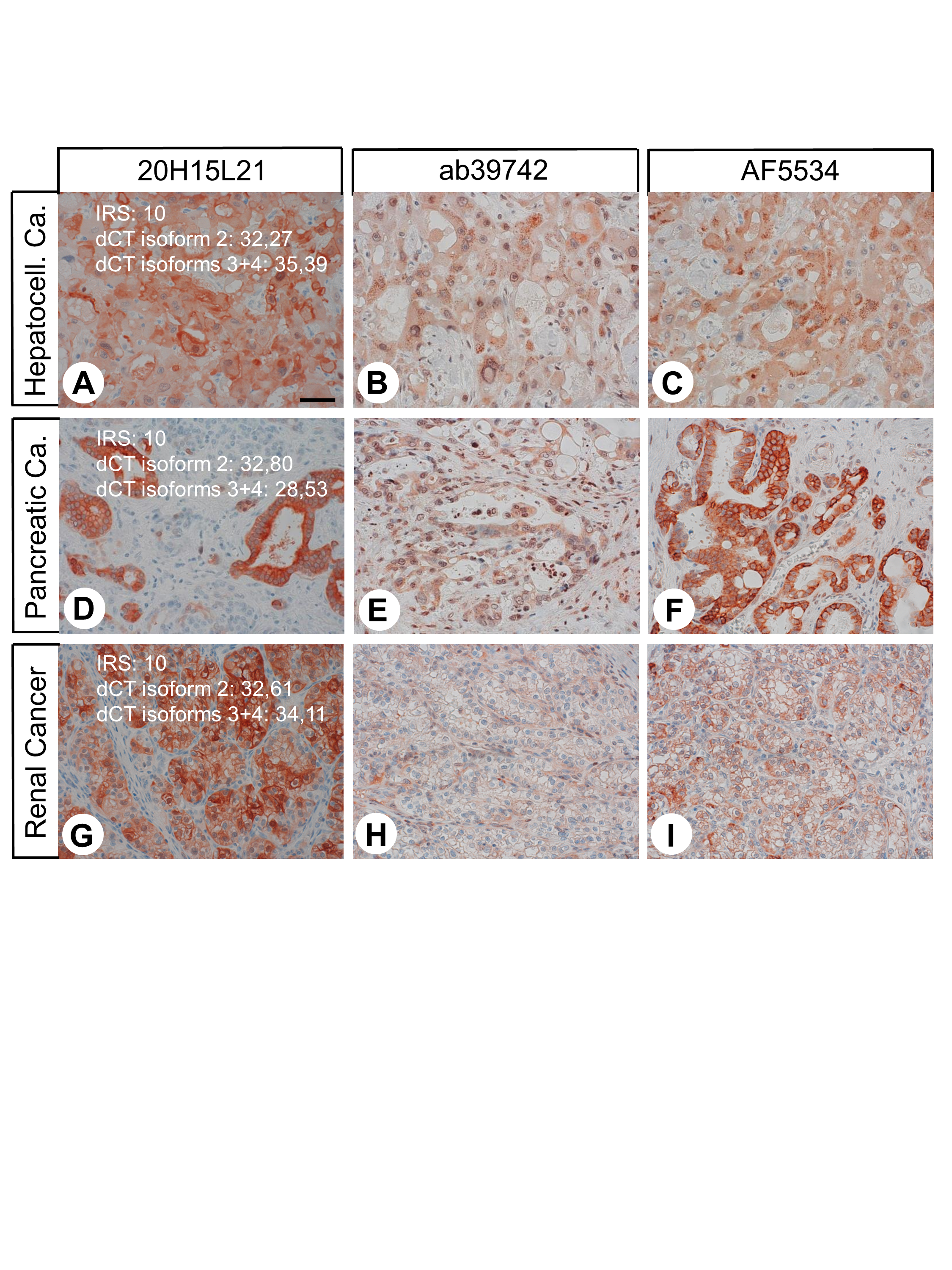

Supplement: Supplementary file 1 [file ijms-23-05191-s001.zip › Supplementary Figure 2.tif]

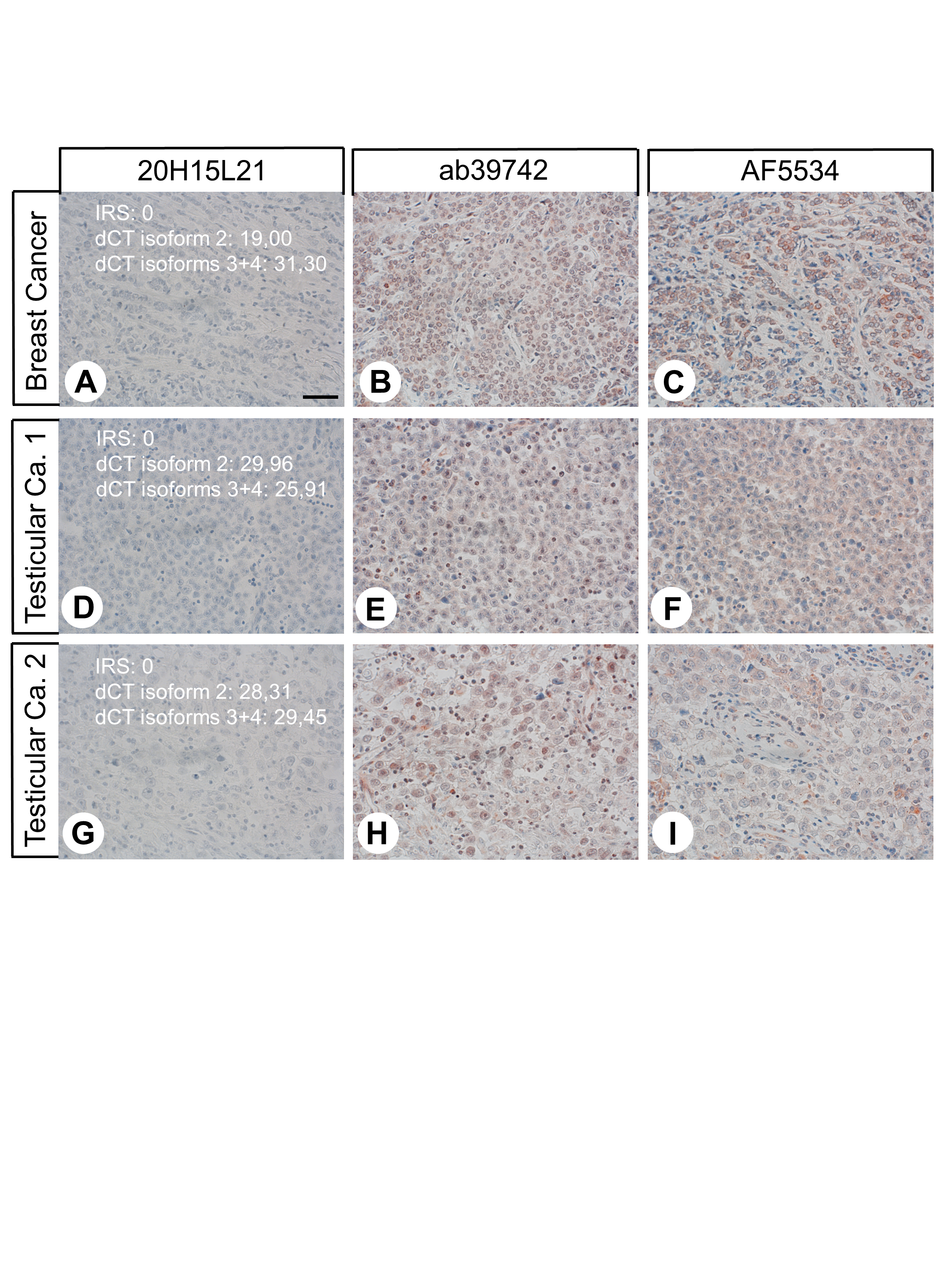

Supplement: Supplementary file 1 [file ijms-23-05191-s001.zip › Supplementary Figure 3.tif]
